# The First Squamous Cell Carcinoma After Kidney Transplantation: A Sentinel Event

**DOI:** 10.1016/j.ekir.2026.106579

**Published:** 2026-05-04

**Authors:** Agavriloaei Bogdan Dumitru, Stephanie Gallitano

**Affiliations:** 1 University of Medicine and Pharmacy “Grigore T. Popa.” Iasi, Romania; 2 Clinical Hospital “Dr. C.I. Parhon” Iaşi, Romania; 3 Dermatology, Columbia University Medical Center, New York, New York, USA; 4 New York-Presbyterian, New York, New York, USA

See Clinical Research on Article 106522

Despite major advances in immunosuppressive strategies and transplant survival, malignancy remains a dominant cause of late mortality in kidney transplant recipients, with cutaneous squamous cell carcinoma (CSCC) representing the most frequent and clinically challenging entity among White patients.^1^

In the article by Crisp *et al.*,^2^ they present data from the COAST study, a multicenter retrospective cohort including 136 kidney transplant recipients diagnosed with a first-ever CSCC across 8 UK transplant centers. Over a median follow-up of 39 months, the outcomes remained poor, with nearly half of the patients developing subsequent CSCC (48.5%) and more than one-fifth dying (23.3%), most commonly because of malignancy. Importantly, the study highlights substantial variability in post-CSCC management, with only 28.7% of patients undergoing immunosuppression reduction and no consistent therapeutic strategy across centers. These findings reinforce previous historical observations while providing contemporary, real-world validation.

In this study, outcomes after index CSCC remained largely unchanged to historical data, with nearly half of patients with CSCC developing another lesion within 3 years and 13.3% developing metastatic disease. There have been advances in immunosuppressive treatment over the past 2 decades, with the recognition that calcineurin inhibitors are implicated in oncogenic risk and SCC development, whereas mTORi and mycophenolate are associated with lower rates of CSCC.^3^ However, as highlighted here and in the literature, specific guidelines for the management of immunosuppressive medication in the setting of malignancy do not exist. The marked heterogeneity in post-CSCC management reflects a state of true clinical equipoise, where clinicians are forced to balance oncologic risk against graft survival in the absence of robust evidence.

These findings support a conceptual shift: rather than representing an isolated dermatological event, the first CSCC should be interpreted as a clinical marker of increased systemic vulnerability. In this context, it may act as a prognostic inflection point, identifying transplant recipients at heightened risk of recurrent malignancy and premature mortality. As illustrated in Figure 1, this transition is associated with a substantial downstream clinical burden and underscores persistent gaps in current evidence and management strategies.

**Figure 1:**
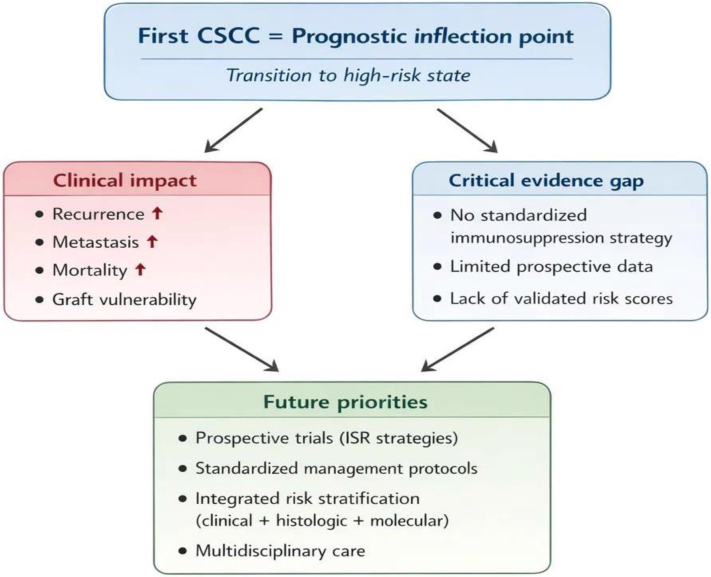
Posttransplant CSCC as a prognostic inflection point and indicator of a critical evidence gap. CSCC, cutaneous squamous cell carcinoma.

This study has several important strengths that enhance its clinical relevance. The multicenter design, spanning 8 transplant centers, provides a broader and more representative perspective than previous single-center reports, which have traditionally dominated this field. The use of a contemporary cohort reflects more current immunosuppressive practices, although we recognize that patients may have been transplanted in the mid-2000s, thereby crossing different treatment approaches. The inclusion of detailed clinicopathological data, including histopathologic risk stratification and management patterns, further strengthens the analysis. Notably, the external validation of outcomes in an independent international cohort supports the robustness and generalizability of the findings.

However, several limitations should be acknowledged. The retrospective design inherently introduces the potential for selection bias and unmeasured confounding, precluding causal inference. The lack of standardized management protocols across centers, though informative in highlighting real-world variability, complicates the interpretation of treatment effects. In addition, key variables such as cumulative ultraviolet exposure and smoking status were incompletely captured, which may have influenced risk estimation. The studied population was overwhelmingly White, and the results of this study cannot be extrapolated to transplant recipients of different races. Finally, as an observational study, the analysis does not evaluate the efficacy of specific interventions, leaving unresolved the critical question of optimal post-CSCC management strategies.

Several key clinical messages emerge from these findings. First, the occurrence of a first CSCC should be viewed as a clinical turning point rather than an isolated dermatologic event. It signals a transition to a higher-risk state, with substantial implications for subsequent cutaneous malignancy, and overall survival. Second, these data highlight the urgent need for improved risk stratification. The identification of high-risk features at the time of the first CSCC, including high-grade histopathology, multiple index lesions, history of basal cell carcinoma, smoking, and male sex, offers a framework for early identification of patients who may benefit from intensified surveillance and tailored intervention. Third, the study underscores a need for guidelines for chemoprophylaxis and strategies for the reduction of immunosuppression after diagnosis of first CSCC. A multidisciplinary approach with dermatology and transplant offers an opportunity for targeted intervention. However, the implementation of these strategies remains highly variable in clinical practice, reflecting the absence of standardized guidelines, concerns regarding graft rejection, drug-related toxicity, and the lack of prospective data to guide decision-making. Notably, the study highlights substantial intracenter and intercenter variation in both immunosuppression modulation and chemoprevention use, further underscoring the lack of standardized management pathways. In addition, certain interventions, particularly topical therapies, may be underreported in routine clinical documentation, complicating the assessment of their real-world implementation. This variability highlights a persistent gap between theoretical management strategies and real-world practice.

Crisp *et al.* highlight a concerning stagnation in outcomes following posttransplant CSCC, despite advances in transplant care. This study serves as a wake-up call, underscoring the urgent need for evidence-based, standardized strategies to improve long-term prognosis. Without a shift toward structured risk stratification and targeted intervention, this high-risk population will continue to face substantial and largely preventable morbidity and mortality. The time to move from observation to intervention is now.

## Disclosure

All the authors declared no competing interests.
